# Decoupling of morphological disparity and taxic diversity during the adaptive radiation of anomodont therapsids

**DOI:** 10.1098/rspb.2013.1071

**Published:** 2013-10-07

**Authors:** Marcello Ruta, Kenneth D. Angielczyk, Jörg Fröbisch, Michael J. Benton

**Affiliations:** 1 School of Life Sciences, University of Lincoln, Lincoln LN2 2LG, UK; 2 Field Museum of Natural History, Chicago, IL 60605–2496, USA; 3 Museum für Naturkunde, Leibniz Institute for Research on Evolution and Biodiversity, Berlin 10115, Germany; 4 School of Earth Sciences, University of Bristol, Bristol BS8 1RJ, UK

**Keywords:** Anomodontia, bottleneck, Dicynodontia, disparity, diversity, Permian extinction

## Abstract

Adaptive radiations are central to macroevolutionary theory. Whether triggered by acquisition of new traits or ecological opportunities arising from mass extinctions, it is debated whether adaptive radiations are marked by initial expansion of taxic diversity or of morphological disparity (the range of anatomical form). If a group rediversifies following a mass extinction, it is said to have passed through a macroevolutionary bottleneck, and the loss of taxic or phylogenetic diversity may limit the amount of morphological novelty that it can subsequently generate. Anomodont therapsids, a diverse clade of Permian and Triassic herbivorous tetrapods, passed through a bottleneck during the end-Permian mass extinction. Their taxic diversity increased during the Permian, declined significantly at the Permo–Triassic boundary and rebounded during the Middle Triassic before the clade's final extinction at the end of the Triassic. By sharp contrast, disparity declined steadily during most of anomodont history. Our results highlight three main aspects of adaptive radiations: (i) diversity and disparity are generally decoupled; (ii) models of radiations following mass extinctions may differ from those triggered by other causes (e.g. trait acquisition); and (iii) the bottleneck caused by a mass extinction means that a clade can emerge lacking its original potential for generating morphological variety.

## 1. Introduction

Mass extinctions affect clades differently. Some disappear completely; others are seemingly unaffected; yet others survive but may experience a loss in taxic diversity and/or a decrease in their range of morphological variety (i.e. disparity). Likewise, the survivors of mass extinctions exhibit a variety of responses. Some decline and go extinct (‘dead clade walking’ [1]), whereas others persist at reduced taxic diversity or undergo new phases of diversification. Surviving clades that rediversify are said to have passed through an evolutionary bottleneck [2]. This is the macroevolutionary analogue of a population bottleneck in which a species is reduced to a small number of individuals, such that its restricted gene pool affects later phases of evolution. In this context, it is therefore logical to ask whether bottlenecks induced by mass extinctions produce similar consequences. In particular, we seek to establish whether clade-level extinctions produce analogous founder effects that potentially limit the disparity and/or the ecological diversity of surviving groups, even if their taxic diversity subsequently increases.

The end-Permian mass extinction event (EPME; about 252 Ma), a biological crisis in which only 4–20% of marine species survived [3], generated a macroevolutionary bottleneck. Its impact on terrestrial organisms is the subject of ongoing research, but it is now clear that its magnitude on land was nearly as profound as in the marine realm [4]. Despite the fact that the terrestrial fossil record is significantly patchier than the marine record, the quality of the terrestrial vertebrate data is generally regarded as adequate to address macroevolutionary questions [5]. Therefore, tetrapod data offer considerable potential for macroevolutionary analyses in the context of this major biological crisis for several reasons. First, although some recent studies found a close relationship between the diversity of terrestrial vertebrates and various proxies for rock availability (e.g. number of formations, number of localities, rock volume and outcrop area per time interval [6–16]), analyses of sampling through the EPME showed that the high diversity of tetrapods before the EPME and their sharp decline in the earliest Triassic are not controlled by rock availability or collecting efforts [15–18]. Second, the global stratigraphic standards for the Permian and Triassic have improved substantially. Primary evidence for correlation of certain terrestrial and marine units has been fixed and tested by application of new magnetostratigraphic and radioisotopic methods [3,7,11,16–19]. Third, the complex anatomy of terrestrial vertebrates provides a rich source of characters, and many groups have been subjected to intensive taxonomic revision. Fourth, repeated and continuing large-scale cladistic studies based on extensive taxon/character matrices have resulted in well-vetted phylogenetic trees. Taxonomic groups that straddle the Permo–Triassic boundary (PTB) are eminently suitable for analyses of clade dynamics in the aftermath of the EPME. Here, we focus on anomodont therapsids—a highly diverse clade of herbivorous stem-group mammals—as a case study for in-depth analysis of post-EPME recovery in the terrestrial realm.

Anomodonts showed a wide range of body sizes and ecological adaptations, including terrestrial, semi-aquatic, fossorial and arboreal forms [20–22]. Members of the most speciose anomodont subclade, the Dicynodontia, exhibited caniniform tusks in the upper jaw, a turtle-like beak and stocky bodies with short limbs and tails. Conversely, basal anomodonts had highly divergent morphologies, including tree-climbing and superficially lizard-like species. Although diverse and abundant in the Late Permian, anomodonts were strongly affected by the EPME: only two *Lystrosaurus* species (*L. curvatus* and *L. maccaigi*) are known to have crossed the PTB [23,24], although time-calibrated phylogenies imply that a minimum of three other anomodont lineages must have survived [25]. *Lystrosaurus* is an example of a ‘disaster taxon’ [17,26] owing to its cosmopolitan distribution and very high abundance in the immediate aftermath of the EPME, representing 73% of all vertebrate specimens in the earliest Triassic of South Africa [27].

First documented in the Middle Permian, anomodonts diversified rapidly and steadily, achieving a peak in taxic diversity (41 species) during the latest Permian [7,28]. They suffered a severe decline during the EPME, resulting in low diversity during the Early Triassic, but rebounded in the Middle Triassic before undergoing a final decline in the Late Triassic. This diversity pattern is consistent with an evolutionary bottleneck, because several major lineages went extinct at the EPME and all Triassic species but three are part of a single lineage dating back to the Permian (Dicynodontoidea).

Using character-based analyses of morphological disparity [29], we investigate four possible scenarios about the potential effects of the EPME bottleneck on anomodonts. (i) Taxic diversity and morphological disparity were correlated throughout the group's history. The bottleneck would have caused a temporary decline in disparity followed by a rebound, mirroring the temporal trend in taxic diversity changes. (ii) Taxic diversity and morphological disparity were correlated during the initial radiation of anomodonts but became decoupled after the bottleneck. The loss of several lineages at the EPME would constrain morphological variation in anomodonts as a whole, even when their taxic diversity began to increase during the post-extinction recovery. (iii) Taxic diversity and morphological disparity were decoupled during the initial anomodont radiation but correlated after the bottleneck. The bottleneck would drive subsequent trends in disparity and taxic diversity simultaneously (similar to scenario (i)). (iv) Taxic diversity and morphological disparity were decoupled throughout anomodont history. The bottleneck in taxic diversity at the EPME would be expected to show little or no impact on temporal trends in morphological disparity (similar to scenario (ii); for discussions of the relationships between diversity and disparity, see [30]).

## 2. Material and methods

### (a) Anomodont phylogeny

There has been considerable interest in the phylogeny of anomodonts over the past decade. A recently published, overarching study [31] provides a comprehensive summary of all previous research and presents a new, refined and expanded analysis of anomodont interrelationships (see electronic supplementary material, figure S1), including 87 taxa and 163 characters (20 continuous and 143 discrete). Both Permian and Triassic taxa are included, altogether covering approximately 70% of known species-level anomodont diversity [7]. The selected tree in the electronic supplementary material, figure S1 is one of the two equally parsimonious trees recovered in the primary analysis of the matrix in [31]. These trees differ exclusively in the resolution of relationships among three taxa—*Elph*, *Interpresosaurus* and *Katumbia*. The tree topology was used to delimit nine groups (g1–g9; electronic supplementary material, figure S1) for the purpose of evaluating disparity changes across major levels of anatomical organization in anomodont evolution.

### (b) Multi-variate treatment of pairwise taxon distances

The matrix in [31] provides the basis for analyses of disparity and morphospace occupation. These analyses quantify morphological differences only, that is, disparity in the conventional sense of the word without any functional or ecological interpretations of the results [32,33]. The matrix was converted to generalized pairwise Euclidean distances (see electronic supplementary material, dataset S1), which were subjected to principal coordinates (PCo) analysis. Disparity was quantified using two range- and two variance-based metrics obtained from the PCo scores (coordinates) of taxa (see electronic supplementary material, dataset S2) on the first 10 PCo axes, following well-established protocols [29]. The results based on the root products of ranges and variances are extremely similar to those based on the sums of ranges and variances. Therefore, we focus on the two sums only.

We also used a distance-based metric—distance from the founder [34]—to investigate models of morphospace occupation, both by major anomodont groups and through time. The calculations were based on the generalized Euclidean distances, and *Biseridens* was treated as the ‘founder’ taxon. The distance from the founder is simply the mean generalized Euclidean distance of taxa (in any given group or time interval) from *Biseridens*.

### (c) Patterns of taxon distribution in morphospace

To assess the significance of separation among major groups of anomodonts in morphospace (where groups represent either taxonomic or temporal taxon sets), we used non-parametric tests, namely a non-parametric multi-variate analysis of variance (npMANOVA; [35]) and an analysis of similarities (ANOSIM; [36]) carried out on the PCo scores of taxa along the first 10 PCo axes. In both tests, we assessed the significance level of group separation via 9999 random permutations. With each permutation, the taxa and their associated PCo scores were sampled randomly and reassigned to groups based on the proportions in which taxa appear in the original groups of interest (either taxonomic or temporal). The statistics of association (*F* in npMANOVA; *R* in ANOSIM) for the original groups was compared to the statistic values obtained from random sampling routines. We chose the Euclidean distance as the distance measure for both tests. Finally, we used Bonferroni correction for multiple comparisons in all post hoc tests of the significance of each pairwise comparison between groups (see electronic supplementary material, dataset S5).

### (d) Analyses of diversity and disparity through time

We assessed the statistical dependence (and associated significance) between diversity and disparity by time interval in two slightly different ways. First, for each of the two disparity metrics—sum of ranges and sum of variances—we quantified their rank–order correlations with diversity using Spearman's *ρ* and Kendall's *τ* correlation coefficients. Analyses were conducted in ‘R’ using codes supplied by Dr Graeme T. Lloyd, downloadable from the following site: http://www.graemetlloyd.com/methgd.html. For each correlation test, we used both unrarefied and rarefied median disparity values (see electronic supplementary material, dataset S3). Each of these two categories of values was correlated with the number of taxa per interval. We examined both the total recorded anomodont diversity for each interval (6, 17, 45, 54, 7, 34, 26 and 8 taxa, respectively, in t1–t8) and the number of taxa per interval that were used in the phylogeny (5, 14, 31, 40, 7, 18, 14 and 6 taxa, respectively, in t1–t8) [7,15,16]. For each correlation test, we reported the strength of the correlation and its permutational probability (see electronic supplementary material, dataset S6).

Our second approach is similar to the previous one, but applies generalized differencing to disparity values and diversity counts [13,37]. The generalized differencing method removes trends in time series, and eliminates autocorrelation by calculating the differences between the values in any two adjacent intervals, accounting for the strength of autocorrelation in adjacent intervals [11,13,37]. Once again, the strength and significance of correlations were quantified for all combinations of diversity counts (i.e. total number of anomodont taxa versus number of taxa in the phylogeny) and disparity values (i.e. rarefied versus unrarefied median values of the sum of ranges and the sum of variances). Finally, we also correlated the unrarefied sums of ranges and variances with the total number of major lineages (i.e. groups g1–g9) present in each time interval (2, 3, 6, 6, 2, 4, 3 and 2 lineages, respectively, in t1–t8; electronic supplementary material, dataset S6).

## 3. Results

### (a) Morphospace occupation

**Figure 1.**
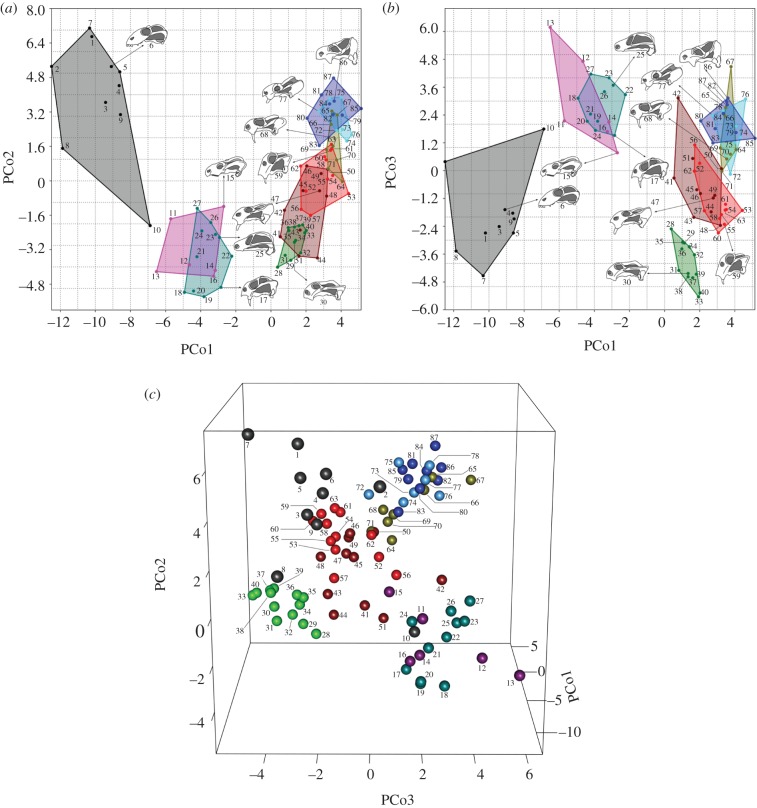
Patterns of anomodont distribution in morphospace. (*a*) Two-dimensional plot on PCo axes 1 and 2; (*b*) two-dimensional plot on PCo axes 1 and 3; in both plots, colour-coded convex hulls delimit taxa in groups g1–g9 and (*c*) three-dimensional plot using PCo axes 1–3, colour-coded according to the scheme in the two-dimensional plots. Group notations and colour codes: g1, basal anomodonts (dark grey); g2, endothiodonts (dark magenta); g3, emydopoids (cyan); g4, cryptodonts (green); g5, ‘*Dicynodon*’-grade taxa (brown); g6, lystrosaurids (red); g7, dinodontosaurids plus shansiodontids (lime); g8, kannemeyeriids (light blue); g9, stahleckeriids (dark blue). Taxon identification numbers: 1, *Biseridens*; 2, *Anomocephalus*; 3, *Patranomodon*; 4, *Suminia*; 5, *Otsheria*; 6, *Ulemica*; 7, *Galepus*; 8, *Galechrius*; 9, *Galeops*; 10, ‘*Eodicynodon*’ *oelofseni*; 11, *Eodicynodon oosthuizeni*; 12, *Colobodectes*; 13, *Lanthanostegus*; 14, *Chelydontops*; 15, *Endothiodon*; 16, *Pristerodon*; 17, *Diictodon*; 18, *Eosimops*; 19, *Prosictodon*; 20, *Robertia*; 21, *Emydops*; 22, *Dicynodontoides*; 23, *Kombuisia*; 24, *Myosaurus*; 25, *Cistecephalus*; 26, *Cistecephaloides*; 27, *Kawingasaurus*; 28, *Keyseria benjamini*; 29, *Daqingshanodon limbus*; 30, *Oudenodon bainii*; 31, *Tropidostoma*; 32, *Australobarbarus*; 33, *Odontocyclops*; 34, *Idelesaurus*; 35, *Rhachiocephalus*; 36, *Kitchinganomodon*; 37, *Aulacephalodon*; 38, *Pelanomodon*; 39, *Geikia locusticeps*; 40, *Geikia elginensis*; 41, *Elph*; 42, *Interpresosaurus*; 43, *Katumbia*; 44, *Gordonia traquairi*; 45, *Delectosaurus*; 46, *Vivaxosaurus trautscholdi*; 47, *Dicynodon lacerticeps*; 48, *Dicynodon huenei*; 49, *Daptocephalus leoniceps*; 50, *Dinanomodon gilli*; 51, *Peramodon amalitzkii*; 52, *Jimusaria sinkiangensis*; 53, *Syops vanhoepeni*; 54, *Euptychognathus bathyrhynchus*; 55, taxon ‘TSK 2’; 56, *Sintocephalus alticeps*; 57, *Basilodon woodwardi*; 58, *Lystrosaurus curvatus*; 59, *Lystrosaurus declivis*; 60, *Lystrosaurus murrayi*; 61, *Lystrosaurus maccaigi*; 62, *Kwazulusaurus shakai*; 63, *Lystrosaurus hedini*; 64, *Turfanodon bogdaensis*; 65, *Dinodontosaurus*; 66, *Dolichuranus*; 67, *Rechnisaurus*; 68, *Tetragonias*; 69, *Vinceria*; 70, *Shansiodon*; 71, *Rhinodicynodon*; 72, *Angonisaurus*; 73, *Xiyukannemeyeria*; 74, *Uralokannemeyeria*; 75, *Parakannemeyeria*; 76, *Rabidosaurus*; 77, *Kannemeyeria simocephalus*; 78, *Kannemeyeria lophorhinus*; 79, *Sinokannemeyeria*; 80, *Placerias*; 81, *Moghreberia*; 82, *Rhadiodromus*; 83, *Wadiasaurus*; 84, *Stahleckeria*; 85, *Sangusaurus*; 86, *Jachaleria*; 87, *Ischigualastia*.

Several aspects of the distribution of anomodonts in morphospace (figure 1; using the first three PCo axes) are of interest. (i) The basal anomodonts (dark grey taxa in figure 1) occupy an extensive region of morphospace that is distinct from the generally smaller regions occupied by each of the major groups of dicynodont anomodonts. (ii) The Permian dicynodonts (dark magenta, most cyan, green, brown and most red taxa in figure 1) occupy a region of morphospace intermediate between those of the basal anomodonts and of the Triassic dicynodonts (most lime, light blue and dark blue taxa). (iii) The Triassic (in particular, Middle and Late Triassic) dicynodonts occupy fairly restricted and sometimes overlapping areas of morphospace. These qualitative patterns are corroborated by the results of npMANOVA and ANOSIM (see electronic supplementary material, dataset S5). Both analyses reject, respectively, the null hypotheses of similar variances (npMANOVA: *F* = 13.5; *p* = 0.0001) and of equal medians and ranges for within-group ranked dissimilarities among major groups (ANOSIM: *R* = 0.6531; *p* = 0.0001). For both analyses, two pairwise post hoc comparisons are non-significant, namely between endothio donts (dark magenta in figure 1) and emydopoids (cyan) and between kannemeyeriids (light blue) and stahleckeriids (dark blue). The latter comparison corroborates the observation that the derived Triassic groups overlap in morphospace. In the case of ANOSIM, a further non-significant comparison occurs between the basal anomodonts (dark grey) and the endothiodonts.

### (b) Disparity

**Figure 2.**
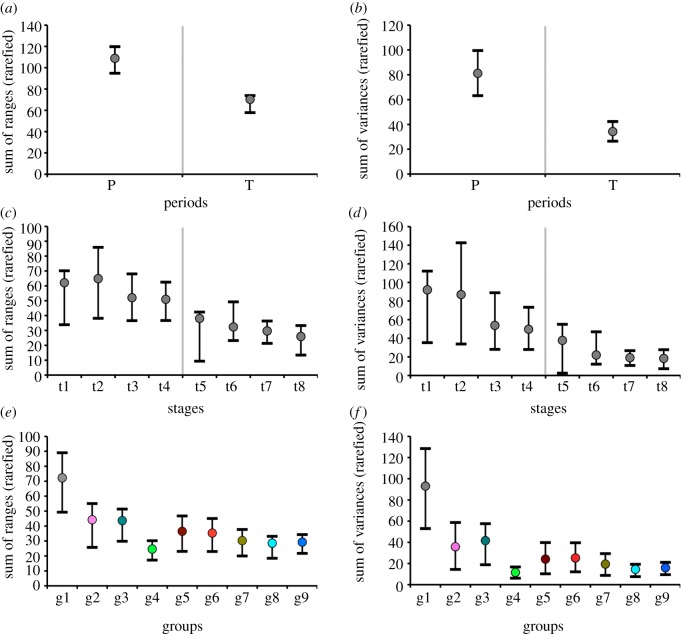
Rarefied median disparity values and associated confidence intervals, based on the sum of ranges (*a*,*c*,*e*) and the sum of variances (*b*,*d*,*f*). (*a*,*b*) Disparity in Permian (P) and Triassic (T) taxa; (*c*,*d*) disparity of taxa assigned to time intervals t1–t8; interval abbreviations: t1, Roadian–Wordian; t2, Capitanian; t3, Wuchiapingian; t4, Changhsingian; t5, Induan–Olenekian; t6, Anisian; t7, Ladinian; t8, Carnian–Norian and (*e*,*f*) disparity of taxa assigned to groups g1–g9 (see figure 1 for group notations and colour codes). Vertical grey bars mark the Permian–Triassic boundary. (Online version in colour.)

The Permian anomodonts are significantly more disparate than their Triassic relatives (see figure 2*a*,*b* and electronic supplementary material, S2*a*,*b*). In addition, Permian and Triassic taxa have significantly different distributions (unequal variances) in morphospace (npMANOVA: *F* = 10.9; *p* = 0.0001). However, we note that the distances within the Permian and Triassic groups do not differ significantly from the distances between these two groups (ANOSIM: *R* = 0.07476; *p* = 0.0655; electronic supplementary material, dataset S5).

Disparity calculations by stage-level time bins (rarefied to the number of taxa present in the least-diverse time bin) paint a more nuanced picture of changes in disparity through time. With the sum of ranges (measuring amount of morphospace occupied; figure 2*c*), we observe an initial slight disparity increase in the late Middle Permian, followed by declines during the rest of anomodont history. With the sum of variances (measuring dispersion of taxa around group centroids; figure 2*d*), we observe a continued decline from an initial high during the Middle Permian to the extinction of the whole group in the Late Triassic. Importantly, disparity during the earliest Triassic stages does not differ significantly (based on overlap among confidence intervals) from disparity in most previous and succeeding stages, although the earliest Triassic was a time of particularly low taxic diversity. These results hold true for the unrarefied sum of variances and, in part, for the unrarefied sum of ranges (see electronic supplementary material, figure S2*c*,*d*). With the latter metric, however, we observe two disparity increases (i.e. between Roadian–Wordian and Capitanian, and between Induan–Olenekian and Anisian), a significant drop at the PTB, and steady decreases between remaining adjacent time intervals. Although the unrarefied sum of ranges exhibits a different pattern from its rarefied counterpart, this results mostly from the significant drop at the PTB, whereas the overall trend of decreasing disparity remains almost unaltered (with the two exceptions noted above). Global tests of separation among groups of taxa assigned to the eight time intervals return significant results, both for npMANOVA (*F* = 7.351; *p* = 0.0001) and for ANOSIM (*R* = 0.258; *p* = 0.0001), although several pairwise comparisons among time intervals are non-significant (electronic supplementary material, dataset S5).

Disparity calculations by major groups (once again rarefied to the number of taxa in the smallest group) show a consistent pattern in terms of both amount of morphospace occupation (figure 2*e*) and degree of taxon dispersal (figure 2*f*). One group, cryptodonts, always emerges as being the least disparate of all. From the root to the apex of the anomodont tree (see electronic supplementary material, figure S1), the three earliest-diverging groups—namely basal anomodonts (g1), endothiodonts (g2) and emydopoids (g3)—are consistently more disparate than all other groups, namely cryptodonts (g4), *Dicynodon*-grade taxa (g5), lystrosaurids (g6), dinodontosaurids/shansiodontids (g7), kannemeyeriids (g8) and stahleckeriids (g9). The unrarefied profile of disparity changes by major group (see electronic supplementary material, figure S2*e*,*f*) shows similar patterns to its rarefied counterpart (figure 2*e,f*).

### (c) Diversity and disparity correlations

**Figure 3.**
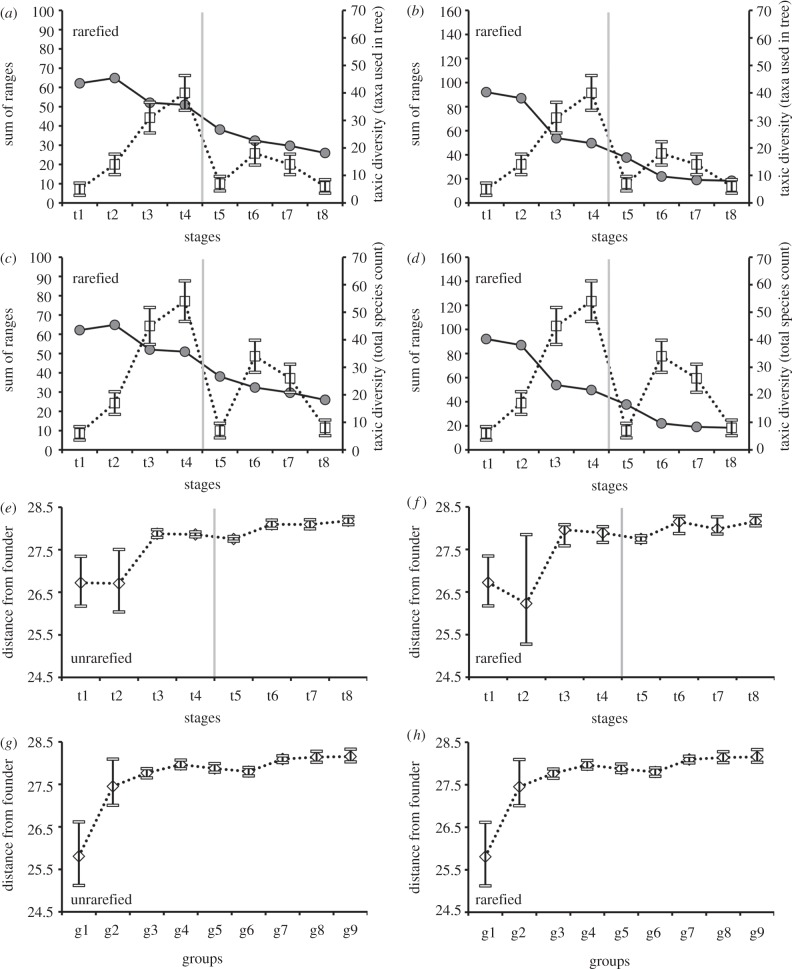
(*a*–*d*) Comparisons between anomodont disparity (rarefied median values, grey circles) and diversity (white squares) through time intervals t1–t8; the disparity values are for the sum of ranges (*a*,*c*,*e*,*g*) and the sum of variances (*b*,*d*,*f*,*h*); the error bars around the diversity values are calculated as ±√*N*, where *N* is the number of taxa in any given interval; (*a*,*b*) comparisons based on the number of taxa present in the phylogeny; (*c*,*d*) comparisons based on the total number of known anomodont taxa. (*e*–*h*) Distance from the founder (white rhombs), expressed as the average generalized Euclidean distance of taxa from *Biseridens* (‘founder’ taxon); (*e*,*f*) average distance of taxa binned by time intervals; (*g*,*h*) average distance of major groups. Vertical grey bars mark the Permian–Triassic boundary.

**Figure 4.**
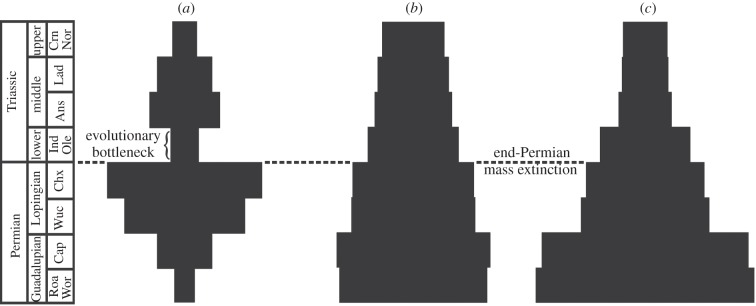
Spindle diagrams highlighting the bottleneck effect of the end-Permian extinction on diversity and disparity. The fine subdivisions of the stratigraphic time scale on the left represent time intervals t1–t8. In each diagram, the widths of the bars are drawn in dimension-less units and proportional to the number of taxa (left diagram) and to the mean disparity values (middle and right diagrams). (*a*) Diversity counts through time based on the number of taxa present in the phylogeny; (*b*) rarefied disparity through time using the median value of the sum of ranges and (*c*) rarefied disparity through time using the median value of the sum of variances.

Anomodont taxic diversity (measured using both the taxa actually included in the phylogeny and the total number of known taxa [7]) increases throughout the Middle and Late Permian, but declines significantly as the PTB is crossed (see figures 3*a*–*d* and 4; electronic supplementary material, S3). It then rebounds during the Middle Triassic before declining again in the Late Triassic. This pattern differs strikingly from that observed for disparity, which generally shows a steady decline over time. Disparity and taxic diversity are very weakly and non-significantly correlated in the vast majority of cases, regardless of which of a wide variety of data treatments are considered (the only exception involves the application of generalized differencing to the unrarefied sum of ranges; electronic supplementary material, dataset S6). Our conclusions also hold true when disparity is correlated with the number of lineages (groups g1–g9) present in each time interval (see electronic supplementary material, figure S4). As in the case of taxic diversity, we found no significant correlations between disparity and number of lineages (see electronic supplementary material, dataset S6) in the vast majority of cases (the sole exception being generalized differencing of the unrarefied sum of ranges).

### (d) Distance from the founder

The average distance of taxa from *Biseridens* is shown in figure 3*e–h*, and calculated through time (figure 3*e*,*f*) and by major groups (figure 3*g*,*h*). With reference to the unrarefied plots (figure 3*e*), the distance from the founder is almost unaltered from Roadian to Capitanian, before a rapid increase is observed at the Capitanian–Wuchiapingian transition, followed by a plateau-like trend until the Induan–Olenekian (only a negligible decrease is observed across the PTB). A new, slightly higher plateau level is attained through the remaining part of the Triassic. The plot of the unrarefied distance from founder for major groups highlights the rapid and steep increase in average distance across the basal anomodont–endothiodont–emydopoid transition (figure 3*g*). Most major groups of dicynodonts are characterized by comparable values, although dinodontosaurids, shansiodontids, kannemeyeriids and stahleckeriids show slightly higher values than other dicynodonts.

## 4. Discussion

### (a) Macroevolutionary bottleneck

Taken together, our results allow us to reject scenarios (i) (diversity and disparity were correlated throughout anomodont history) and (ii) (diversity and disparity were correlated during the initial radiation of anomodonts but became decoupled after the bottleneck) for the potential bottleneck effect of the EPME on anomodonts. Clearly, disparity and diversity were neither correlated throughout the entire history of this clade (scenario (i)), nor is there evidence for a correlation between these two variables (or between disparity and number of major lineages) in the Permian that subsequently broke down in the Triassic (scenario (ii)). Instead, rarefied anomodont disparity declines steadily over time, whereas taxic diversity fluctuates, with two episodes of increase alternating with two episodes of decrease. This pattern is consistent with scenario (iv), which posits that taxic diversity and disparity may be controlled by different factors. Scenario (iii) applies only to the unrarefied sum of ranges in the post-EPME history of the group and may be partly expected given the sensitivity of this metric to sample size, and the fact that the majority of Triassic anomodonts appear superficially very similar. Therefore, although the EPME represents a bottleneck in terms of anomodont taxic diversity, it does not seem to have had a strong effect on broad-scale patterns of morphological variation throughout the evolution of this group (figure 4).

Similarly, and perhaps more surprisingly, the ecological opportunities occurring in the aftermath of the EPME apparently were not enough to alter the trend of declining disparity in anomodont history. Despite their sheer diversity, numerical abundance and wide geographical distribution, these therapsids appear to have been constrained in the amount of morphological novelty that they could generate. A potential example of such constraints can be found in the evolution of the distinctive feeding system of the dicynodonts. Early in the group's history, a series of sweeping changes to the skull, including the addition of a novel muscle mass and the origination of a highly distinctive jaw joint morphology [38–41], resulted in a propalinal (i.e. fore–aft) sliding motion of the lower jaw during mastication. However, subsequent changes to the skull–jaw articulation system tended to be minor. Even when certain Triassic lineages re-emphasized an orthal (i.e. up-and-down) motion of the lower jaw, they did so by changing the skull proportions and slightly altering the shape of the articular surface of the jaw joint (such that extensive fore–aft sliding at the joint translated into an orthal instead of a propalinal motion at the anterior tip of the jaw) rather than by evolving fundamentally new skull and/or jaw features [42].

The distance from the founder (see, especially, figure 3*e*,*g*) highlights the generally conservative nature of ‘higher’ dicynodonts relative to basal anomodonts and primitive dicynodonts. The rapidly increasing values of this metric across groups g1–g3 point to the major structural transition from basal taxa to dicynodonts and correspond to the temporal increase of the average distance from the founder early in anomodont history. After the initial increase, however, the overall structural differences among dicynodonts tend to show very limited excursions when expressed in terms of distance from the founder. Simply expressed, an ‘average’ dicynodont taxon from any given time interval or major group is not more similar/dissimilar to a basal anomodont than an ‘average’ dicynodont taxon from any other time interval or group. This implies that dicynodonts may have been constrained from evolving any fundamentally new, distinct morphologies despite variations in ecological opportunities and fluctuations in diversity.

### (b) Phylogeny and disparity signal

Recent work [33] suggests that much of the disparity signal provided by cladistic datasets has phylogeny as one of its main sources. In light of this, we might expect anomodont disparity to correlate with the number of groups present at any time, i.e. being higher when several clades co-occur, and lower when only a few clades are present and/or when most taxa belong to just a single clade (because members of a single clade would be relatively similar owing to their descent from a recent common ancestor). The overall temporal trend in anomodont diversity shows a declining number of clades over time. As an example, groups such as endothiodonts, emydopoids, cryptodonts and dicynodontoids are present in the Late Permian, whereas Late Triassic anomodont faunas are dominated by stahleckeriids. Therefore, a declining disparity trend might be predicted. However, the reality is more complex.

The group's history starts with the low-diversity/high-disparity basal anomodonts. Although taxic diversity increases rapidly with the appearance of new clades during the Middle and early Late Permian, disparity increases only at the Roadian–Wordian to Capitanian transition (in the case of both unrarefied and rarefied sum of ranges), but begins to decrease immediately thereafter, despite the continued addition of major anomodont clades. At the EPME, disparity continues to decrease except in the case of the unrarefied sum of ranges. The pattern described for this metric mirrors the post-EPME trend in diversity changes, whereby an initial diversity increase is observed at the Induan–Olenekian to Anisian transition, and a novel steady decrease occurs thereafter. Taken together, our findings suggest that, at least for anomodonts, tree topology (i.e. phylogenetic relatedness) may not strongly (or exclusively) affect the overall trends in disparity changes.

In summary, taxic diversity and morphological disparity are decoupled in anomodont history, with a macroevolutionary bottleneck in one (taxic diversity) apparently not affecting long-term trends in the other (disparity). This observation is significant because it implies that studies that consider taxic diversity or morphological disparity in isolation may miss important features of a clade's history, such as those that may be revealed by more holistic approaches. This is especially relevant in analyses of large-scale evolutionary diversifications, when simple taxon counts may provide little or no insight into the timing of acquisition of important adaptations or the impact of these on modalities of clade expansion. At the same time, it will be important to investigate the interplay between diversity and disparity in other vertebrate groups, in order to determine whether the anomodont pattern is typical or atypical during mass extinctions.
